# *Shewanella algae* bacteraemia in a patient with a chronic ulcer after contact with seawater on vacation in Turkey: A case report from a German maximum-care hospital

**DOI:** 10.1016/j.nmni.2022.101016

**Published:** 2022-08-29

**Authors:** Cara Symanzik, Jutta Esser, Niels Pfennigwerth, Christoph Reuter, Jan Bronnert, Matthias Grade

**Affiliations:** 1) Institute for Interdisciplinary Dermatological Prevention and Rehabilitation (iDerm) at Osnabrück University, Osnabrück, Germany; 2) Department of Dermatology, Environmental Medicine and Health Theory, Osnabrück University, Osnabrück, Germany; 3) Laboratory Medical Practice Osnabrück, Georgsmarienhütte/Osnabrück, Germany; 4) National Reference Centre for Multidrug-Resistant Gram-Negative Bacteria, Department of Medical Microbiology, Ruhr-Universität Bochum, Bochum, Germany; 5) Department of General Surgey and Abdominal Surgery, Specialized Abdominal Surgery and Proctology, Christian Hospital of Quakenbrück, Quakenbrück, Germany; 6) Department of Pneumology and Infectiology, Christian Hospital of Quakenbrück, Quakenbrück, Germany; 7) Department of Gastroenterology, General Internal Medicine and Infectiology, Christian Hospital of Quakenbrück, Quakenbrück, Germany

**Keywords:** Bacteraemia, Emerging pathogen, rare pathogen, Shewanellaceae, *Shewanella algae*, Skin and soft tissue infection (SSTI)

## Abstract

After seawater baths in Antalya, Turkey, a 55-year-old man suffered from *Shewanella algae* bacteraemia. Imported/travel-related *S. algae* infections should be kept in mind, also in usually rather cold geographical areas, as patterns of seawater-associated bacilli infections might change due to warming of seawater caused by climate change.

## Main text

*Shewanella algae* is the predominant species in the family of Shewanellaceae causing human infections. Its' ability to produce toxins can result in severe skin and soft tissue infections followed by systemic infections [1]. Infections are seen especially in immunocompromised patients with pre-existing damage of cutaneous integrity [2,3].

We report a case of a 55-year-old man, who suffered from a chronic ulcer in the area of his right upper ankle joint due to a deep venous thrombosis three years ago. The ulcer was regularly under surgical supervision and apparently closed and sealed by incrustation. In early September 2021, the man visited Antalya, Turkey, where he took seawater baths. The temperature of air and water was up to 32°C and 25°C, respectively.

**Fig. 1:**
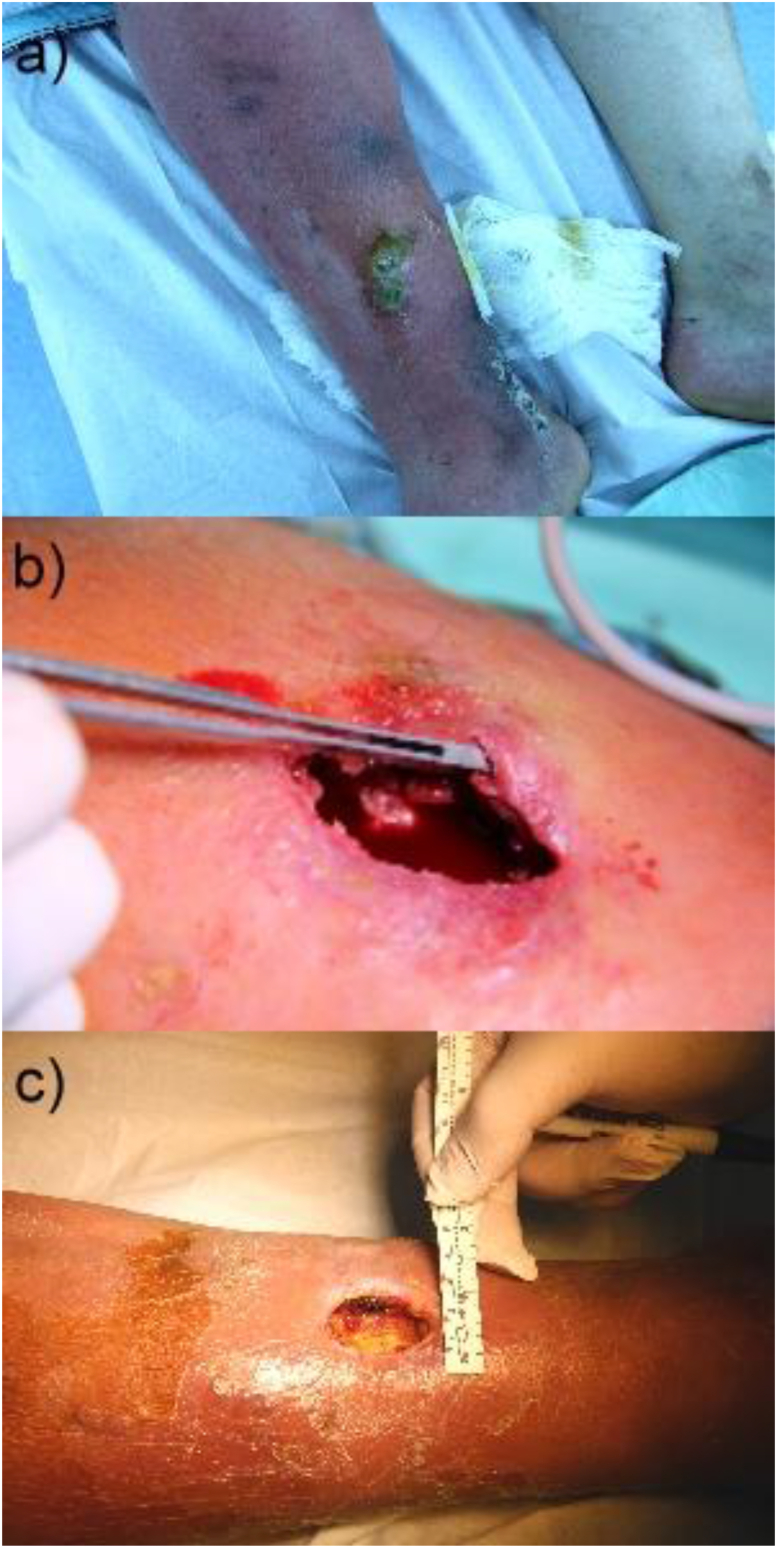
*Shewanella algae* infection in a patient with a chronic ulcer in the area of the right upper ankle joint. a) Continuous worsening of the condition at the patient’s right leg. b) Surgical treatment of the ulcer. Two abscesses had to be removed and a mass of necrotic tissue was drained, leaving behind two deep defects down to the periosteum. Under continuous surgical care with initial use of a vacuum pump, the local situation improved slowly. c) Further course (with a view down to the bone) under continuous surgical debridement and supervision.

Four weeks after vacation, he felt ill (fever, malaise, pain in the ulcerous leg) and presented himself in our emergency unit. He was in poor general condition; his body temperature indicated 38.9°C and the right lower leg was deeply red (Fig. 1). The working diagnosis ‘erysipelas’ was presumed. Treatment with benzylpenicillin (3 × 10 Mio IU) was started immediately. Two days later, a pair of two initially drawn blood culture sets was positive for *S. algae* examined by Maldi-TOF MS. Samples from vesicles in the area of the pre-existing ulcer showed growth of *S. algae* as unique pathogen. Using EUCAST-non-species-related breakpoints (Vitek II microdilution), sensitivity to broad-spectrum ß-lactam-antibiotics (except carbapenems) and fluorchinolones was revealed. A propensity to carbapenem-insensibility was seen due to an intrinsic carbapenemasis out of the OXA-55-family (Ambler Class D), typical for *S. algae* [4], proven by whole genome sequencing. The detected OXA-55-variant hasn't yet got a unique designation. A hitherto unknown allele of the gyrB gene impedes assignment to existing multilocus sequence typing.

Therapy was changed to piperacillin/tazobactam (3 × 4.5 g IV) combined with ciprofloxacin (3 × 400 mg IV). After 3 weeks, a treatment change to cotrimoxazole (2 × 960 mg) per os for additional 6 weeks was continued. A few days after starting the appropriate pharmacological therapy, the systemic infection resolved. However, the condition at his right leg continuously worsened, probably due to an additional effect of toxin-production, as described for *S. algae*. More than 3 months later, there is residual damage and ongoing need for surgical debridement and supervision. A retirement process was started, as the patient cannot conduct professional activities anymore.

*S. algae* are distributed in marine habitats with water temperatures above 13°C, optimally 20°C [5]. Reports classify *S. algae* as emerging pathogen; occurrence in rather colder regions of the world is mainly attributed to warming of seawater [6]. Most human *S. algae* infections were reported in warmer areas [3]. It is, however, conceivable that we may face similar unusual disease patterns in the future [5].

Given the potentially grave consequences of *S. algae* infections, we present this case to facilitate awareness. Particular attentiveness should be devoted to patients with chronic skin or soft tissue diseases who had contact to seawater or consumed raw seafood within the last four weeks and face sudden and unclear worsening of their general condition. Also, it is of importance to consider blood cultures and local cultures in all febrile patients and before antibiotic treatment. This could easily be overlooked, but provides the only way to correct diagnosis of an unusual pathogen, hence saving suffering, time, and healthcare costs. Due to contemporary travel habits, *S. algae* should be kept in mind, also in rather cold geographical areas, and restricted sensitivity to carbapenems should be considered.

## Author contributions (CRedIT)

**Cara Symanzik:** Conceptualization, Visualization, Writing - original draft; Writing - review & editing; **Jutta Esser:** Conceptualization, Investigation, Resources, Writing - review & editing; **Niels Pfennigwerth:** Investigation, Writing - review & editing; **Christoph Reuter**: Conceptualization, Investigation, Resources, Writing - review & editing; **Jan Bronnert:** Conceptualization, Investigation, Resources, Writing - review & editing; **Matthias Grade:** Conceptualization, Investigation, Resources, Writing - review & editing. All authors have read and approved the final submitted version of the manuscript.

## Transparency declaration

None declared. This work did not receive any external funding.

## Data availability statement

Data available on request from the authors.
